# Personalizing the first dose of FSH for IVF/ICSI patients through machine learning: a non-inferiority study protocol for a multi-center randomized controlled trial

**DOI:** 10.1186/s13063-024-07907-2

**Published:** 2024-01-11

**Authors:** Nuria Correa, Jesus Cerquides, Josep Lluis Arcos, Rita Vassena, Mina Popovic

**Affiliations:** 1 Universitat Auntonoma de Barcelona (UAB), Bellaterra, Barcelona, 08193 Spain; 2 Artificial Intelligence Research Institute, IIIA (CSIC), Campus de la UAB, Bellaterra, Barcelona, 08193 Spain; 3 Clinica Eugin-Eugin Group, Carrer de Balmes 236, Barcelona, 08006 Spain; 4 Present Address: Fecundis, Baldiri i Reixac, Barcelona, Spain

**Keywords:** Controlled ovarian stimulation, FSH, IVF, Machine learning, Decision support system, Artificial intelligence

## Abstract

**Background:**

Adequately selecting the initial follicle-stimulating hormone (FSH) dose during controlled ovarian stimulation (COS) is key for success in assisted reproduction. The objective of COS is to obtain an optimal number of oocytes to increase the chances of achieving a pregnancy, while avoiding complications for the patient. Current clinical protocols do achieve good results for the majority of patients, but further refinements in individualized FSH dosing may reduce the risk of poor ovarian response while also limiting the risk of ovarian hyperstimulation syndrome (OHSS) risk. Models to select the first FSH dose in COS have been presented in literature with promising results. However, most have only been developed and tested in normo-ovulatory women under the age of 40 years.

**Methods:**

This is a randomized, controlled, multicenter, single blinded, clinical trial. This study will be performed in 236 first cycle in vitro fertilization (IVF) and/or ICSI (intracytoplasmic sperm injection) patients, randomized 1:1 in two arms. In the intervention arm, the dose of FSH will be assigned by a machine learning (ML) model called IDoser, while in the control arm, the dose will be determined by the clinician following standard practice. Stratified block randomization will be carried out depending on the patient being classified as expected low responder, high responder, or normo-responder. Patients will complete their participation in the trial once the first embryo transfer result is known. The primary outcome of the study is the number of metaphase II (MII) oocytes retrieved at ovarian pick up (OPU) and the hypothesis of non-inferiority of the intervention arm compared to the control. Secondary outcomes include the number of cycle cancelations (due to low response or no retrieval of mature oocytes), risk of ovarian hyperstimulation syndrome (OHSS), and clinical pregnancy and live birth rates per first transfer.

**Discussion:**

To our knowledge, this is the first randomized trial to test clinical performance of an all-patient inclusive model to select the first dose of FSH for COS. Prospective trials for machine learning (ML) models in healthcare are scarce but necessary for clinical application.

**Trial registration:**

ClinicalTrials.gov, NCT05948293. Registered on 14 July 2023.

**Supplementary information:**

The online version contains supplementary material available at 10.1186/s13063-024-07907-2.

## Administrative information

Note: the numbers in curly brackets in this protocol refer to SPIRIT checklist item numbers. The order of the items has been modified to group similar items (see http://www.equator-network.org/reporting-guidelines/spirit-2013-statement-defining-standard-protocol-items-for-clinical-trials/).

**Table:** 

Title {1}	Personalizing the first dose of FSH for IVF/ICSI patients through machine learning: a non-inferiority study protocol for a national multi-center randomized controlled trial
Trial registration {2a} and {2b}	NCT05948293. Registered on the 14th of July 2023.
Protocol version {3}	Version 2. 2nd February 2023
Funding {4}	Nuria Correa is supported by and industrial scholarship “Doctorat Industrial” funded by the Generalitat de Catalunya [DI-2019-24], by the project CI-SUSTAIN funded by the Spanish Ministry of Science and Innovation [PID2019-104156GB-I00] and by intramural funding provided by Clínica Eugin-Eugin Group. This work is supported by intramural funding provided by Clínica Eugin-Eugin Group.
Author details {5a}	Nuria Correa^1,2,3^, Jesus Cerquides^2^, Josep Lluis Arcos^2^, Rita Vassena^3^, Mina Popovic^3^
	1: Universitat Autonoma de Barcelona (UAB), 08193 Bellaterra, Barcelona, Spain.
	2: Artificial Intelligence Research Institute, IIIA (CSIC), Campus de la UAB, 08193 Bellaterra, Barcelona, Spain.
	3: Clinica Eugin-Eugin Group, Carrer de Balmes 236, 08006 Barcelona, Spain.
Name and contact information for the trial sponsor {5b}	Clínica Eugin (Euvitro S.L.U. B-61663506). C/ Balmes, 236 08006 Barcelona (Spain). Phone: +34 933221122. e-mail: investigacion@eugin.es
Role of sponsor {5c}	The sponsor designed the study, processed and interpretated the data, and wrote this manuscript.

## Introduction

### Background and rationale {6a}

Controlled ovarian stimulation (COS) is a key step for assisted reproduction and fertility treatment. During COS, clinicians prescribe different medications to stimulate the ovaries to produce several mature oocytes [1]. Oocytes are retrieved from the ovaries during the oocyte pickup (OPU) and later fertilized with sperm in the laboratory. Controlling the number of mature or metaphase II (MII) oocytes is critical for success. Ultimately, a higher number of MII oocytes increases the chance of pregnancy [2, 3]. However, treating a patient with the aim of retrieving as many MII oocytes as possible also increases the discomfort and costs of the treatments and the risk of ovarian hyperstimulation syndrome (OHSS), a potentially life-threatening medical condition resulting from an excessive response to stimulating hormones.

The first step in COS involves the selection of an adequate first dose of follicle-stimulating hormone (FSH). This first dose of FSH is important, as it recruits available ovarian follicles to grow. Once follicles are recruited, little can be done to modify the number of oocytes that will be retrieved [2]. A balance must be struck between recovering sufficient mature oocytes to ensure optimal clinical outcomes, while minimizing the risks to the patient. Assigning an appropriate starting dose of FSH thus constitutes an essential clinical decision in the personalization of treatment.

In routine clinical practice, the choice of starting FSH dose is based on patient characteristic, such as her age, body mass index (BMI) and ovarian reserve, amongst others. Yet, despite clinical experience and current evidence-based practice, criteria to accurately select the starting dose of FSH for all patients have not been entirely identified. Accordingly, patients with similar characteristics (or even the same patient treated at different timepoints) may have different, often unexpected, outcomes following the same treatment, possibly displacing them from the optimal range of oocytes (10 to 15 [3, 4]).

Machine learning (ML) models that capture medical experience registered in clinical historical data have been devised in an effort to reduce the extremes of ovarian response and automate the personalization of FSH dose selection. Two good examples include nomograms that use patient age, anti-Müllerian hormone (AMH), or antral follicle count (AFC) and basal endogenous FSH to tailor the first dose of FSH for COS [5, 6]. The nomogram by La Marca et al. (2012) has also been tested prospectively in a randomized controlled trial (RCT) [7]. The authors observed that in the intervention arm, a higher number of patients fell within the optimal range of MII oocytes, while fewer had poor outcomes. Howles et al. developed a model based on multivariate regression [8]. The model was subsequently tested in an RCT study [9]. While similar pregnancy rates were achieved compared to clinical practice, the authors observed a reduction in OHSS cases when using the model. Finally, another randomized trial was performed for a model developed specifically for FSH delta [10]. Dose allocation by the model resulted in more patients within the target response (8–14 oocytes), less excessive responses ($\ge$ 15 oocytes in the high AMH stratum), and less poor responses (< 4 oocytes in the low AMH tier), when compared to a non-personalized-dosing control group. However, no differences in pregnancy and live birth rates were observed.

While these approaches report clinically relevant outcomes, they were developed and tested using data from normo-ovulatory patients under the age of 40. Ultimately, patients for whom correct FSH dosing is most critical were excluded. To account for a more comprehensive IVF/ICSI patient population, we developed and trained an inclusive FSH dosing model named IDoser [11].

IDoser includes age, body mass index (BMI), AMH levels, and AFC as predictors. It was developed and trained while incorporating available clinical evidence encoded as rules during parameter optimization. The objective of IDoser is to optimize the number of oocytes retrieved during ART treatment, achieving an appropriate balance defined as 10–15 oocytes, as suggested by literature ([3, 4]). When compared to real-life clinical practice, the IDoser performed significantly better (*p *< 0.05), showing an expected 11.6% increase in the proportion of patients who were closer to the optimal range of oocytes [11, 12]. In this study, we have specifically chosen a non-inferiority trial design with the specific goal of refining and standardizing the FSH dosing model in clinical practice. This approach is fundamentally different from attempting to prove the superiority of the new model. Instead, our focus is on demonstrating that our newly developed FSH dosing model maintains at least the same level of efficacy as the existing clinical standard. Opting for a non-inferiority trial is particularly beneficial in this context, as the current standard for FSH dosing is effective, and our goal is to offer an alternative that provides more standardized dosing guidelines and reduces variability in patient outcomes, particularly in the number of oocytes retrieved, without sacrificing treatment efficacy. By aiming to align closely with the high-quality care standards already in place, this methodology is well-suited to our objectives of enhancing and streamlining the FSH dosing process. Furthermore, the non-inferiority trial’s requirement for a smaller sample size aligns well with the practical constraints of clinical research, making it an appropriate choice for assessing our FSH dosing model.

### Objectives {7}

This RCT tests the hypothesis that the performance of IDoser in prescribing the initial dose of FSH during COS is not inferior to the performance of a clinician in the average number of mature oocytes retrieved. Secondary objectives include the effect of IDoser on number of cycle cancelations (due to low response or no retrieval of oocytes), number of cases with OHSS risk, clinical pregnancy, and live birth per first transfer.

### Trial design {8}

The trial design is a single-blinded RCT with two arms with a 1:1 allocation ratio, comparing outcomes following FSH dose selection by IDoser and dosing based on standard clinical practice.

The hypothesis that will be tested is whether the performance of IDoser selecting the first dose of FSH is non-inferior to the standard clinical protocol in regards to the average number of mature oocytes recovered per group. The difference in the number of MII that is regarded to be clinically significant is 2 MII. Our decision to consider a difference of two MII oocytes as clinically significant is based on empirical evidence and clinical relevance. As only 30 to 40% of inseminated mature oocytes develop into blastocysts [13, 14], the choice of two MIIs strikes a balance between statistical significance and clinical practicality, while taking into account the consequent impact on the success of fertility treatments. If the average number of MII in the intervention group is inferior in 2 or more units, IDoser will be considered inferior to the standard clinical practice.

## Methods: participants, interventions, and outcomes

### Study setting {9}

This is a randomized, single-blinded, multicenter, clinical trial. It will be carried out in two distinct centers in Spain, both of which are part of the same IVF group.

### Eligibility criteria {10}

Eligibility criteria for patient recruitment in this study are rigorously established and will be assessed by the clinician during the pre-treatment consultation. The inclusion criteria for participants are as follows: individuals aged 18 years or older, but not exceed 50 years of age; first autologous conventional IVF and/or ICSI cycles, irrespective of the underlying causes of infertility; the use of FSH on the first day of stimulation (which can be combined with luteinizing hormone, LH). Exclusion criteria include: natural cycles (without COS); and cycles in which FSH is not measured in international units (IU).

### Who will take informed consent? {26a}

Participants will be enrolled in the trial only after receiving comprehensive information about the study, being provided with a written consent form by medical personnel, and formally agreeing to participate by signing the form. They will be informed of the study during by their consulting physician following the recommendation of an IVF/ICSI treatment. Patients considering participation will receive a comprehensive consent form from the medical team following the clinical consultation. This form comprehensively outlines the nature of the study, what participation entails, and the rights of the participants. The signed document will be retrieved prior to starting COS.

### Additional consent provisions for collection and use of participant data and biological specimens {26b}

Not applicable; data collected in this study will not be used for any ancillary studies.

## Interventions

### Explanation for the choice of comparators {6b}

Patients in the control group will be prescribed their first dose of FSH by the clinician in accordance with standard clinical practice.

Both participating centers adhere to a uniform clinical practice protocol, ensuring consistency in patient treatment and data collection across the trial. In both participating centers, eligible patients will undergo controlled ovarian stimulation, which can be induced with either recombinant FSH (Gonal®, Merck-Serono, Spain; Bemfola®, Gedeon Richter Iberica, Spain) or highly purified human menopausal gonadotropin or hMG (Menopur®, Ferring, Spain; Meriofert®; IBSA; Spain). The first dose of FSH, administered on the second day of the menstrual cycle, is routinely determined by expert physicians for specific patients depending on their age, ovarian reserve markers (AMH levels, AFC), and BMI. Pituitary inhibition will be performed with a GnRH antagonist (Orgalutran®, Organon, Netherlands), and ovulation will be triggered when $\ge$ 3 follicles of $\ge$ 17 mm diameter are observed, using 250 mcg of choriogonadotropin alfa (Ovitrelle®; Merck-Serono, Spain) or 0.3 mg of Triptorelin (Decapeptyl®, Ipsen Pharma, France) in cases with a risk of OHSS. Following oocyte retrieval, the sperm sample will be prepared for conventional IVF and/or ICSI. Fertilized oocytes will be cultured in vitro and transferred either fresh or frozen. The preferred clinical strategy is single embryo transfer on day 5 of embryonic development, although double embryo transfer and/or day 3 embryo transfer may be performed in specific cases.

### Intervention description {11a}

Patients in the intervention arm will be prescribed the first dose of FSH by IDoser that will take into account the age of the patient, BMI, AFC, and AMH. These data will be retrieved from the patient’s clinical file after their first visit to the clinic, after providing the patient with informed consent documentation.

### Criteria for discontinuing or modifying allocated interventions {11b}

Participants can withdraw from the study at any time and for any reason or no reason. The reason for withdrawal will be recorded if patients choose to disclose this information. Failure to administer the allocated FSH dose and discontinuation of IVF/ICSI for medical reasons will also result in withdrawal. Participants will be informed immediately regarding their withdrawal from the trial, if either an error in dose administration is detected or for a medical reason. If an error in FSH administration or medical reason is detected, the participant’s treatment will be interrupted or allowed to continue based on the clinical judgment of the healthcare provider. The data of withdrawn patients obtained during their participation in the study will be included in the study analysis. Withdrawn patients will not be replaced.

### Strategies to improve adherence to interventions {11c}

To ensure adherence to the prescribed intervention, specifically the administration of the initial dose of FSH, a designated study nurse will oversee and verify the correct dosing for each patient. Post-intervention, the treatment process will proceed as per routine clinical practice, without additional interventions made by the study team. The research team will ensure that the patient has adequate follow-up post-intervention in order to retrieve all relevant outcome data. This follow-up is in line with the standard care protocol for all IVF/ICSI patients, encompassing post-OPU medical visits, daily communication with the IVF laboratory team about the progress of in vitro culture, scheduling of the embryo transfer (whether fresh or frozen), and monitoring of any subsequent pregnancy. All relevant data will be tracked and recorded in the electronic medical records as part of routine clinical practice.

### Relevant concomitant care permitted or prohibited during the trial {11d}

Outside of first FSH dose allocation, the COS and IVF/ICSI treatments will be under control of the assigned clinician of the participant, as per routine practice.

### Provisions for post-trial care {30}

Care post-trial will follow routine practice and will be controlled by the assigned clinician for each participant.

In accordance with the Spanish legislation regarding clinical trials with medicines (Real Decreto 223/2004 6th of July), the sponsor of the study will subscribe an insurance covering the sponsor, investigator, collaborators, and center. This will cover any contingencies in the event of deleterious consequences for participants.

### Outcomes {12}

The primary efficacy criterion will be the number of mature, MII oocytes retrieved at OPU. This treatment outcome is the closest to the intervention and has a clear impact on IVF/ICSI cycle success. Secondary efficacy endpoints will include cycle cancelations (due to poor response or in the event that no mature oocytes are retrieved), OHSS risk, clinical pregnancy, and live birth per first transfer.

The following variables will be analyzed:

- Demographic variables
  - Age of the patient (years)
  - AFC
  - BMI (kg/m$$^2$$)
  - AMH (ng/ml)
  - Center of treatment
- COS outcome variables
  - Number of MII oocytes
  - Number of cumulus-oocyte complexes (COCs)
  - Estradiol at last ultrasound assessment (pg/ml)
  - Number of follicles $\ge$ 11mm at last ultrasound check
  - OHSS risk ([yes/no]; risk is considered as present if estradiol > 5000 pg/ml and or $\ge$ 18 follicles $\ge$ 11mm at last ultrasound check)
  - OPU cancelation ([yes/no]; present if COS is stopped prior to OPU)
  - Cycle cancelation ([yes/no]; present if no MII oocytes are retrieved at OPU)
- Pregnancy outcome variables
  - Clinical pregnancy ([yes/no]; present if positive fetal heart beat is observed at 7th week of gestation) per first embryo transfer
  - Live birth per first embryo transfer ([yes/no])

### Participant timeline {13}

Participant schedule for enrollment, interventions, assessments, and trial relevant visits can be visualized in Table 1.

**Table 1:** SPIRIT flowchart of enrollments and assessments. Detailed timing for relevant events for participants during the randomized trial

	Study period						
	Enrollment	Allocation	Post-allocation				Close-out
Time point	$\varvec{-t}_{\varvec{1}}$	$\varvec{0}$	$\varvec{t}_{\varvec{1}}$	$\varvec{t}_{\varvec{2}}$	$\varvec{t}_{\varvec{3}}$	$\varvec{t}_{\varvec{4}}$	$\varvec{t}_{\varvec{5}}$
**Enrollment:**							
**Eligibility screen**	✕						
**Informed consent**	✕						
**Allocation**		✕					
**Interventions:**							
***FSH dose selection by model***			✕				
***FSH dose selection by clinician***			✕				
**Assessments:**							
***Baseline variables***	✕	✕					
***Outcome variables***				✕	✕	✕	✕

A detailed description of the timepoints reflected in the figure is as follows:

- $-t_1$: First appointment with the clinician where information on IVF/ICSI treatment is relayed to the patient. Information on the trial is communicated to the patient, and informed consent documentation is handed over to eligible patients. If any of the baseline variables needed for IDoser to function (age, BMI, AFC, and AMH) are not available at this time, steps are put in place to obtain relevant information for the next appointment. These can include petition of blood tests for AMH level results, or echography for AFC assessment.
- $t_0$: Appointment with the clinician where the signed informed consent form is retrieved. Patient is included if all baseline variables are available. Once included, the participant is allocated to either the IDoser FSH selection group or control group. The FSH dose is prescribed to the participant.
- $t_1$: First day of the COS protocol. Participant will self-administer the prescribed dose of FSH.
- $t_2$: Last ultrasound appointment prior to OPU. First outcome variables are registered (OHSS risk and OPU cancelation).
- $t_3$: Day of OPU. Further outcome variables are registered (number of COCs recovered, number of MII recovered and cancelation after OPU).
- $t_4$: Appointment to evaluate the presence of a fetal heart beat at the 7th week of gestation to establish whether pregnancy is achieved after first embryo transfer in current IVF/ICSI cycle. Presence or absence of clinical pregnancy is registered.
- $t_5$: End of study, considered after outcome on live birth after first embryo transfer is obtained. Presence or absence of live birth achieved is registered.

Baseline variables include all necessary variables required for IDoser, including age of the patient, BMI, AFC, and AMH levels.

### Sample size {14}

A sample size calculation was performed to establish the number of participants required for the study. To determine a statistically significant difference equal or greater to 2 MII oocytes, 118 subjects are necessary in each group (*n *= 236), accepting an alpha risk of 0.05 and a beta risk of 0.2 in a one-sided test. We estimate a mean common standard deviation of 5.84 (as per the observational data included during development and validation of IDoser) and anticipate a drop-out rate of 10%.

### Recruitment {15}

All potential participants will be informed about the study by their clinician prior to undergoing IVF/ICSI treatment.

## Assignment of interventions: allocation

### Sequence generation {16a}

Stratified block randomization will be carried out depending on whether the patient is expected to be a poor responder (AMH < 1.2 ng/ml and AFC < 5, as per POSEIDON criteria [15]), high responder (AMH $\ge$ 3 ng/ml and AFC $\ge$ 15), or expected normo responder (any other case). This will ensure equal distribution of these patient etiologies across both arms. There will be 3 blocks (one for each strata) for every arm. The size of every block has been determined by the population distribution of each mentioned strata during the development and validation of the IDoser to be studied (13$\%$ poor, 15$\%$ high and 72$\%$ normo-responders) [11]. These figures translate to 15 patients in the poor responders group, 18 in the high responders, and 85 in the normo-responders within each arm. The study group (treatment or control) will be randomly assigned using a computer program with a 1:1 allocation ratio.

The 3 randomization lists will be generated using the online software Graphpad (http://www.graphpad.com/quickcalcs/randMenu/). This is a single-blinded trial, in which the patients are blinded to the source of the dose prescribed. Other clinicians apart from the one assigned to the participant, embryologists and part of the research team will also be blinded.

### Concealment mechanism {16b}

Single-blind trial. Participants will not be aware of which arm they have been placed in. Once the random allocation sequences are generated, they will be stored in an electronic data table accessible to the responsible clinician. Once the data for IDoser is introduced into the table, the arm allocation field will be populated with the next free sequence value of the participant strata. Apart from the data coordinator, the research team will also be blinded to participant allocation.

### Implementation {16c}

The allocation sequence will be generated with the Graphpad tool by a member of the research team. Enrollment will be carried out by medical doctors. Once included, participants will receive the prescription for the first FSH injection dose, whether decided by the clinician or IDoser, and self-administer it as indicated by their clinician.

## Assignment of interventions: blinding

### Who will be blinded {17a}

Participants will be blinded to arm allocation. The research team will also be blinded, with the exception of the data coordinator. The medical team, with the exception of the ones assigned to care for the participants, will also be blinded to arm allocation. The embryology laboratory team will also be blinded.

### Procedure for unblinding if needed {17b}

Unblinding for participants will be permissible after their participation in the trial is ended. Additionally, it will be permissible for patients and the medical and embryology team in the event of a medical event that justifies unblinding.

## Data collection and management

### Plans for assessment and collection of outcomes {18a}

All trial data will be collected as per standard clinical practices for IVF/ICSI treatments in the participant clinics.

### Plans to promote participant retention and complete follow-up {18b}

There are no additional plans for retention of participants.

### Data management {19}

Data will be registered both in the electronic data table where allocation is provided and in a separate registry file. In the electronic data base, maximum and minimum value checks will be performed for every variable (if applicable).

### Confidentiality {27}

Participants’ information will be stored in both an electronic data table and registry file in a secure folder with controlled access only accessible to clinicians and the research team involved in the trial. This folder will be located in a secure server under exclusive control of the sponsor. Both the data table and registry file will be password protected. All data will be anonymized. Personal data will be protected according to the Regulation (EU) 2016/679 of the European Parliament and the Council of 27th April 2016 on the protection of natural persons in regards to processing of personal data and on the free movement of such data (General Data Protection Regulation or GDPR). In Spain, in addition to the GDRP, the transposition of this regulation to the national “Ley Orgánica 3/2018, de 5 de diciembre, de Protección de Datos Personales y garantía de los derechos digitales” will apply.

Relevant data will be stored 10 years after the finalization of the trial as per Regulation (EU) 2017/745 from the 5th of April.

### Plans for collection, laboratory evaluation, and storage of biological specimens for genetic or molecular analysis in this trial/future use {33}

Not applicable as no biological specimens will be retrieved during this trial for any genetic or molecular analysis; only data will be registered for the purposes of this specific trial.

## Statistical methods

### Statistical methods for primary and secondary outcomes {20a}

#### Descriptive analysis

Description of all demographic and result variables included in the trial will be provided overall and per study group (mean, standard deviation or SD, *n*, $\%$).

#### Univariable analysis

Differences regarding the number of MII oocytes amongst the groups will be evaluated using Student’s *t* test or Mann-Whitney *U* test (if the distribution is not normal). These tests will also be used to compare FSH doses and COC number.

Regarding all categorical variables (OHSS risk, OPU cancelation, cycle cancelation, clinical pregnancy, live birth), differences between groups will be evaluated using Pearson’s chi-squared. Description of adverse events (if any) will be provided by the study group.

Alpha risk is set at a 5% for the primary analysis, focusing on the difference between MII oocytes retrieved between the two arms of the study. For secondary and subgroup analysis, appropriate statistical adjustment methods will be implemented.

All analyses will be performed using Python version 3.7.6. A *p*-value < 0.05 will be considered as statistically significant.

### Interim analyses {21b}

There is no interim analysis planned for this study.

### Methods for additional analyses (e.g., subgroup analyses) {20b}

Subgroup analysis will be carried out for participants predicted to be low, high, and normo-responders.

### Methods in analysis to handle protocol non-adherence and any statistical methods to handle missing data {20c}

Non-adherence to the trial protocol would imply that the participant has not received the intervention planned. These participants will be considered as having withdrawn from the trial. This per-protocol analysis strategy ensures that our study results accurately reflect the outcomes for those participants who fully adhered to the intervention protocol.

Missing covariates would immediately be considered as exclusion criteria, as they are necessary for the randomization and/or the use of the IDoser.

### Plans to give access to the full protocol, participant-level data, and statistical code {31c}

Fully anonymized participant level data and the statistical code used for this trial will be made available by the corresponding author on reasonable request.

## Oversight and monitoring

### Composition of the coordinating center and trial steering committee {5d}

This is a multicenter trial, where the coordinating center is Clinica Eugin in Barcelona. Daily support for the study is provided by the:

- Principal investigator: supervises the trial and coordinates the study team
- Data coordinator: manages data annotation and data safety and quality
- Research team: includes both principal investigator and data coordinator, together with co-investigators in charge of data outcome analysis
- Medical team: in charge of participant recruitment, handling of informed consent forms, follow-up of participants, and safety monitoring according to protocol

There is no steering committee.

### Composition of the data monitoring committee, its role and reporting structure {21a}

The unblinded data coordinator will be in charge of monitoring safety and quality of data and will report to the principal investigator. The data coordinator is not independent from the sponsor. The research team has no commercial conflict of interest.

### Adverse event reporting and harms {22}

Clinical study participants will be routinely asked about adverse events (quantity and quality) at each study visit. Any adverse event that may occur to the participants of the study must be documented and followed up by the investigator. The event will be documented with the necessary investigations for adequate assessment of causality as established in the document “MDCG 2020-10/1- Safety reporting in clinical investigations of medical devices under the Regulation (EU) 2017/745.” Serious adverse events must be immediately notified to the sponsor, who will be in charge of reporting the events to the ethics committee and the competent authorities. The sponsor must report serious adverse events within 15 days (7 days in case of death or a life-threatening event) using the official serious adverse events notification forms. The sponsor will report the serious adverse events through Eudavigilance-CT.

### Frequency and plans for auditing trial conduct{23}

This trial is subject to external audit independent from investigators and the sponsor, annually. The data coordinator will perform internal audits quarterly, by randomly selecting a subset of participants and crosschecking the electronic data table and informed consents.

### Plans for communicating important protocol amendments to relevant parties (e.g., trial participants, ethical committees) {25}

Any amendment to the trial protocol will be reported to the ethics committee and competent authorities and only applied after their approval.

### Dissemination plans {31a}

Plans to disseminate results and conclusions of the trial include scientific papers and/or congress communications.

## Discussion

The current literature includes several FSH dosing models [5, 6, 8, 10] that when tested prospectively [7, 9, 10] show improvement in outcomes. This includes a reduction in OHSS risk and a higher proportion of patients within the optimal range. Nonetheless, none of these models consider patients over the age of 40 years or non-normo-ovulatory women in their development nor in the respective RCTs. As such, the applicability of current models remains limited. The trial presented would evaluate an all-inclusive, comprehensive FSH model for COS, named IDoser. This kind of trial for interventional ML models is sparse in literature, but necessary for a safe clinical application of any medical device of this nature.

## Trial status

Registered in ClinicalTrials.gov, under identifier NCT05948293, on 14 July 2023. Recruitment has not started yet; it is estimated to start in October 2023 and end October 2024. Current protocol version is version 2 from 2 February 2023.

### Supplementary information

**Additional file 1.** Ethical Committee for Research approval. Signed approval of the current trial protocol by the Ethical Committee for Research of Eugin.

**Additional file 2.** Consent form (Spanish). Original consent form for the current trial protocol approved by the Ethical Committee for Research of Eugin. Redacted in Spanish.

**Additional file 3.** Consent form (English). English version of the consent form for the current trial protocol approved by the Ethical Committee for Research of Eugin.

## Data Availability

Anonymized data will be shared upon reasonable request.

## Abbreviations

IVF: In vitro fertilization
ICSI: Intracytoplasmic sperm injection
MII: Metaphase II
COS: Controlled ovarian stimulation
FSH: Follicle-stimulating hormone
OPU: Oocyte pick-up
OHSS: Ovarian hyperstimulation syndrome
BMI: Body mass index
AMH: Anti-Müllerian hormone
AFC: Antral follicle count
RCT: Randomized controlled trial
LH: Luteinizing hormone
IU: International units
COCs: Cumulus-oocyte complexes
GDPR: General data protection regulation
SD: Standard deviation
