# Supplementary material for: Personalizing the first dose of FSH for IVF/ICSI patients through machine learning: a non-inferiority study protocol for a multi-center randomized controlled trial
Source: Trials. 2024 Jan 11;25:38. doi: 10.1186/s13063-024-07907-2 (PMC10782678; doi:10.1186/s13063-024-07907-2)
Supplement: Supplementary file 1 — Additional file 1. Ethical Committee for Research approval. Signed approval of the current trial protocol by the Ethical Committee for Research of Eugin. [file 13063_2024_7907_MOESM1_ESM.pdf]

CEImeugin
Comité de Ética
de la Investigación con medicamentos

Opinion of the Ethical Research Committee

Dra. Rita Vassena, president of the Comité de Ética de la Investigación con medicamentos (CEIm) EUGIN

CERTIFIES

That this committee has evaluated the following study proposal

TITLE: Randomized investigation with an AI powered medical device to select the first dose of FSH for Controlled Ovarian Stimulation

PROTOCOL:	Version 02	Date: 02/02/2023
HIP/CI GENERAL:	Version 02	Date: 02/02/2023

SPONSOR: EUGIN (EUVITRO S.L.U.) Code: ALGO3

PRINCIPAL INVESTIGATOR: Dra. Mina Popovic

That this Committee has evaluated the sponsor's proposal to carry out the abovementioned study, has reviewed the researcher's modifications to the modifications requested by this Committee (if any), and considers that:

- The study is appropriate, taking into consideration available knowledge.
- The study is designed in accordance with the ethical principles stated in the Declaration of Helsinki developed by the World Medical Association for medical research in human participants, and posterior revisions.
- The procedure for handling of personal data is appropriate and will be done in accordance to:
    - "Ley 14/2006, de 26 de mayo, sobre técnicas de reproducción humana asistida, la Orden SAS 3470/2009"
    - "el Real Decreto 1090/2015 de EECC, por el que se regulan los ensayos clínicos con medicamentos, los Comités de Ética de la Investigación con medicamentos y el Registro Español de Estudios Clínicos"

- The centers and researchers listed on Annex II of this document are considered appropriate.
- The procedure for obtaining informed consent (including the test subject information sheets and informed consents mentioned above) is considered appropriate.

- The compensation provided to the participants is adequate, as are the provisions for compensation for damages that the participant may suffer.
- The procedure provided for the handling of personal data is adequate and is carried out in accordance with the provisions of Regulation (EU) 619/2016 of the European Parliament and of the Council, of April 27, 2016, regarding the protection of natural persons in regarding the processing of personal data and the free circulation of these data and the "Ley Orgánica 3/2018, de 5 de diciembre, de Protección de Datos Personales y garantía de los derechos digitales (LOPDGDD)".

That this Committee's APPROVED the study during the meeting held on the 23/3/2021 (Minutes nº 03).

That the CEIm EUGIN, both in composition and procedures, complies with the guidance on Good Clinical Practice (CPMP/ICH/135/95) as well as with current legislation that govern its functioning, with the "Real Decreto 1090/2015 de EECC, por el que se regulan los ensayos clínicos con medicamentos, los Comités de Ética de la Investigación con medicamentos y el Registro Español de Estudios Clínicos" and the "Decreto 106/2003, de 24 de octubre, por el que se regulan los requisitos y el procedimiento de acreditación de los comités de ética de investigación clínica en Cataluña".

That the composition of CEIm EUGIN is indicated on Annex I, considering that if any Committee member is part of the research team or declares a conflict of interest, they would not have participated neither in the deliberation nor the final decision on the study proposal.

Signed in Barcelona on 08/03/2023

Dra. Rita Vassena
President - CEIm EUGIN
