# Supplementary material for: Personalizing the first dose of FSH for IVF/ICSI patients through machine learning: a non-inferiority study protocol for a multi-center randomized controlled trial
Source: Trials. 2024 Jan 11;25:38. doi: 10.1186/s13063-024-07907-2 (PMC10782678; doi:10.1186/s13063-024-07907-2)
Supplement: Supplementary file 2 — Additional file 2. Consent form (Spanish). Original consent form for the current trial protocol approved by the Ethical Committee for Research of Eugin. Redacted in Spanish. [file 13063_2024_7907_MOESM2_ESM.pdf]

HOJA DE INFORMACIÓN PARA LA PARTICIPANTE

Estudio aleatorizado de un producto sanitario basado en inteligencia artificial seleccionador de la primera dosis de FSH para estimulación ovárica

Estudio promovido por Clínica Eugin y realizado por la Dra. Mina Popovic como investigadora principal.

Antes de que decida si va a participar o no y que firme la hoja de consentimiento, es importante que lea detenidamente la siguiente información y formule todas las preguntas que crea conveniente.

Introducción

Para poder realizar un ciclo de fecundación in vitro, sus ovarios deben ser estimulados usando una medicación hormonal que hace crecer los folículos, dentro de los cuales se hayan los óvulos. De esta forma y tras un número de días de medicación diaria, cuando los folículos tienen la medida considerada adecuada por el profesional médico, se procede a recuperar los óvulos que haya dentro mediante la punción ovárica. La primera dosis de esta medicación determinará cuántos folículos empiezan a crecer en este proceso. Por lo tanto, pautar esta primera dosis en la cantidad adecuada es un paso importante en el tratamiento de fecundación in vitro, ya que una dosis baja puede llevar a recuperar pocos óvulos, y una dosis demasiado alta a un posible riesgo de hiperestimulación ovárica. El protocolo estándar, desarrollado acorde a la evidencia científica y la vasta experiencia del cuerpo médico de la clínica, tiene en cuenta datos como su edad, reserva ovárica y pasados tratamientos si los hubiera, para ajustar esta primera dosis.

Para incorporar aún más personalización y eficiencia al protocolo actual se ha considerado la aplicación de inteligencia articial, que permite analizar grandes cantidades de datos y refinar procesos como la personalización de la dosis de medicación. Este análisis se hace utilizando complejos modelos matemáticos, llamados algoritmos. Recientes publicaciones muestran algoritmos con resultados prometedores en esta tarea, aunque muchos aún no están probados clínicamente y los que lo están no cubren a toda la población.

En Eugin, hemos desarrollado un modelo de inteligencia artificial que ha demostrado, en sus fases de desarrollo y de validación preclínica, la capacidad de mejorar la dosificación en entre un 5,6% y un 32% de los casos.

¿Cómo funciona este sistema en la práctica?

El sistema se divide en dos partes: una plataforma online en la cual el profesional médico cargará la información necesaria para su análisis (edad, índice de masa corporal y datos de reserva ovárica); y

un modelo de inteligencia artificial que analiza estos datos de forma autónoma, reportando la dosis de medicación hormonal recomendada personalizada.

¿Para qué se hace este estudio?

Tras haber cumplido con las validaciones preclínicas y haber comprobado su buen funcionamiento téorico, necesitamos comprobar por primera vez si en la práctica el modelo de inteligencia artificial desarrollado equipara como mínimo nuestra capacidad de identificar la primera dosis ideal de medicación hormonal en pacientes. Para ello, el objetivo de este estudio es comparar el número de óvulos maduros recuperados entre un grupo control y un grupo donde se asigne la primera dosis de medicación con el algoritmo desarrollado.

¿Por qué he sido elegida para participar?

Para poder llevar a cabo este estudio piloto necesitamos de 236 participantes, aleatorizadas en dos grupos (control vs estudio). Usted ha sido elegida porque va a realizar su primer ciclo de fecundación in vitro (FIV) con óvulos propios. Adicionalmente no presenta ninguna de las características de exclusión de este estudio: tratamiento con ciclo natural y/o uso de medicación hormonal no medida en unidades internacioles (UI).

¿En qué consiste mi participación?

Si usted acepta participar en este estudio, será asignada al azar a uno de los dos grupos que vamos a comparar: tratamiento en estudio: asignación de primera dosis de medicación hormonal por parte del algoritmo; o tratamiento habitual (grupo control): asignación de primera dosis hormonal siguiendo el protocolo médico estándar. Esto quiere decir que tiene el 50% de posibilidades de recibir el tratamiento en estudio y 50% de posibilidades de recibir el tratamiento habitual. Los resultados relevantes de su tratamiento serán analizados, desde el número de óvulos recuperados hasta el resultado de embarazo tras finalizar su tratamiento de fecundación in vitro.
Su participación en este estudio acabará cuando se sepa este resultado final. Usted no conocerá su clasificación en grupo de estudio o control.

Para poder realizar los análisis previstos en este estudio necesitaremos acceder a su historia clínica para recoger sus datos demográficos y clínicos.

Su participación en este estudio es completamente voluntaria. Su participación o no participación no tendrá ninguna repercusión en la calidad del tratamiento ni en la atención que recibirá. Además, podrá retirarse del estudio en cualquier momento poniéndose en contacto con la investigadora

principal del estudio en el teléfono o en el correo electrónico indicados al final de este documento, sin tener que dar explicaciones y sin repercusión alguna.
Si acepta su participación y puede ser incluída en este estudio se le ofrecerá una compensación de 350 euros.

¿Qué riesgos y/o beneficios puedo esperar de mi participación?

El algoritmo testado en este estudio ha demostrado en sus fases pre-clínicas una mayor precisión en la asignación de la primera dosis de medicación hormonal que la práctica actual, por lo cual se espera que tenga un comportamiento similar en su fase clínica, aunque no existe garantía de que así sea. Esto comportaría para usted, si es el caso, un aumento en las posibilidades de conseguir un número de óvulos maduros más óptimo para su caso particular. En caso contrario, suponiendo que el algoritmo no sea más preciso que la práctica actual, este posible aumento no se daría, o se podría producir una recuperación de ovocitos menor o mayor de la esperada. Una recuperación menor de óvulos maduros podría disminuir las posibilidades de éxito del tratamiento de fecundación in vitro, y una recuperación mayor podría aumentar el riesgo de hiperestimulación ovárica. En este último caso, se utilizaría el protocolo de descarga adecuado para mitigar el cuadro de hiperestimulación, evitando así, en la gran mayoría de casos, problemas para su salud, pero sin poder realizarse transferencia embrionaria en fresco. Los embriones serían congelados y transferidos en su siguiente ciclo menstrual. Dado el diseño conservador del algoritmo y como se ha evidenciado en los resultados preclínicos, no se espera que los riesgos descritos aumenten por su uso respecto a la práctica clínica habitual.

De conformidad con el Art. 69 del reglamento 2017/745 para los productos sanitarios, el promotor de este estudio tiene cobertura por un seguro o indemnización para las pacientes en caso de cualquier evento adverso grave no esperado.

Conforme al artículo 7 de la Ley 14/2007 de Investigación Biomédica su participación implica la renuncia a cualquier derecho económico o de otro tipo sobre el resultado del estudio.

¿Cuál es la normativa aplicable a este estudio?

Siguiendo las guías éticas internacionales y la legislación vigente, este estudio ha sido aprobado por el Comité de Ética de la Investigación con medicamentos de Eugin y la Agencia Española de Medicamentos y Productos Sanitarios.

Sus datos personales serán registrados de forma estrictamente confidencial, conforme con el Reglamento (UE) 2016/679 de 27 de abril relativo a la protección de las personas físicas en relación

con el tratamiento de sus datos personales (RGPD) y la Ley Orgánica 3/2018 de 5 de diciembre de protección de datos personales y garantía de los derechos digitales (LOPDGDD).

Usted, como titular de sus datos personales, tiene derecho al acceso, modificación, oposición y cancelación de sus datos, así como a limitar el tratamiento de datos incorrectos, solicitar una copia o que sus datos se trasladen a un tercero (portabilidad). Le recordamos que sus datos personales no podrán ser eliminados, aunque deje de participar en este estudio, para garantizar la validez de la investigación; únicamente se conservarán a efectos de este estudio y no se cederán a terceros, salvo por obligación legal o la consecución de un interés legítimo de la compañía, de un tercero o de usted mismo. En cumplimiento del Art. 89 del RGPD, así como de lo dispuesto en la Disposición Adicional 17a de la LOPDGDD, sus datos personales recogidos para el presente estudio estarán sujetos a las garantías adecuadas para sus derechos y libertades y, por lo tanto, serán identificados mediante un código, de manera que no se incluya información que pueda identificarle. Sólo el equipo investigador podrá relacionar dichos datos con usted y con su historia clínica. Por lo tanto, su identidad no será revelada a ninguna otra persona salvo a las Autoridades Sanitarias, cuando así lo requieran o en casos de urgencia médica. Los Comités de Ética de la Investigación, los representantes de la Autoridad Sanitaria en materia de inspección y el personal autorizado por el Promotor, únicamente podrán acceder para comprobar los datos personales, los procedimientos del estudio y el cumplimiento de las normas de buena práctica clínica (siempre manteniendo la confidencialidad de la información). La legitimación de tratamiento será el consentimiento expreso e inequívoco prestado por usted mediante la participación en el presente estudio.

Dentro del marco vigente en materia de investigación biomédica, el Investigador y el Promotor están obligados a conservar los datos recogidos para este estudio al menos 25 años tras su finalización (15 años para EECC con medicamentos).

Posteriormente, su información personal solo se conservará por el centro para el cuidado de su salud y por el promotor para otros fines de investigación científica si usted hubiera otorgado su consentimiento para ello.

En el caso de que tuviéramos que realizar una transferencia de sus datos fuera de la UE a las entidades de nuestro grupo, a prestadores de servicios o a investigadores que colaboren con nosotros, los datos del participante, en cumplimiento del Art. 32 del RGPD, y teniendo en cuenta el estado de la técnica, los costes de aplicación, y la naturaleza, el alcance, el contexto y los fines del tratamiento, así como riesgos de probabilidad y gravedad variables para los derechos y libertades de las personas físicas, se aplicarán medidas técnicas y organizativas apropiadas para garantizar un nivel de seguridad adecuado al riesgo, que en su caso incluya, entre otros: a) la seudonimización y el cifrado de datos personales; b) la capacidad de garantizar la confidencialidad, integridad, disponibilidad y resiliencia permanentes de los sistemas y servicios de tratamiento, c) otras

salvaguardas adicionales tales como contratos u otros mecanismos definidos por las autoridades de protección de datos.

Le informamos de que puede contactar con el delegado de protección de datos de Clínica Eugin a través de dpo@eugin.es si tiene cualquier duda, queja o necesita ejercer alguno de sus derechos mencionados. Así mismo, le informamos de su derecho a dirigirse a la Agencia de Protección de Datos si no quedara satisfecho.

Estamos a su disposición para cualquier consulta por teléfono (+34933221122) o por correo electrónico (mpopovic@eugin.es). Gracias a usted, podemos seguir investigando.

HOJA DE CONSENTIMIENTO INFORMADO

Estudio aleatorizado de un producto sanitario basado en inteligencia artificial seleccionador de la primera dosis de FSH para estimulación ovárica

Estudio promovido por Clínica EUGIN y realizado por la Dra. Mina Popovic como investigadora principal.

Yo, (Nombre y Apellidos): ____

Con documento de identidad/pasaporte número: ______________________________

Edad: ________ años y fecha de nacimiento: _________________________________

Manifiesto que he sido informada del presente estudio y:

- Recibido suficiente información sobre el estudio.
- Leído y entendido la hoja de información que se me ha entregado.
- Podido hacer preguntas sobre el estudio y sobre mis derechos.
- Comprendido que mi participación es voluntaria.
- Comprendido que puedo retirarme del estudio cuando quiera, sin tener que dar explicaciones y sin que esto repercuta en mis cuidados médicos.
- Comprendido que mi participación en el estudio no conlleva ningún perjuicio para mi salud.
- Sido informada de que los datos personales serán protegidos y que los resultados de mi evaluación personal serán estrictamente confidenciales.

En consecuencia, doy mi consentimiento para formar parte de este estudio.

Firma de la participante<br><br>Nombre, apellidos<br><br>Fecha: ___________________________	Firma del facultativo que informa<br><br>Nombre, apellidos, nº de colegiado<br><br>Fecha: ___________________________
