# Supplementary material for: Personalizing the first dose of FSH for IVF/ICSI patients through machine learning: a non-inferiority study protocol for a multi-center randomized controlled trial
Source: Trials. 2024 Jan 11;25:38. doi: 10.1186/s13063-024-07907-2 (PMC10782678; doi:10.1186/s13063-024-07907-2)
Supplement: Supplementary file 3 — Additional file 3. Consent form (English). English version of the consent form for the current trial protocol approved by the Ethical Committee for Research of Eugin. [file 13063_2024_7907_MOESM3_ESM.pdf]

TRIAL INFORMATION SHEET FOR THE PARTICIPANT

Randomized Study of an Artificial Intelligence-Based Medical Device for Selecting the First Dose of FSH for Ovarian Stimulation

Study Sponsored by Eugin Clinic and Conducted by Dr. Mina Popovic as Principal Investigator

Before deciding whether to participate and signing the consent form, it is crucial that you carefully read the following information and ask any questions you may have.

Introduction

To undergo an in vitro fertilization (IVF) cycle, your ovaries must be stimulated using hormonal medication to induce the growth of follicles, within which the eggs are located. After a prescribed number of days of daily medication, when the follicles reach a size considered appropriate by the medical professional, the eggs are retrieved through ovarian puncture. The first dose of this medication determines how many follicles begin to grow in this process. Thus, prescribing this initial dose appropriately is a crucial step in any IVF treatment, as a low dose may lead to the retrieval of few eggs, while an excessively high dose may pose a potential risk of ovarian hyperstimulation. The standard protocol, developed in accordance with scientific evidence and the extensive experience of the clinic's medical staff, considers factors such as your age, ovarian reserve, and past treatments, if any, to adjust this initial dose.

To further enhance personalization and efficiency in the current protocol, the application of artificial intelligence (AI) has been considered. AI allows the analysis of large datasets and the refinement of processes such as the customization of medication dosage. This analysis is conducted using complex mathematical models known as algorithms. Recent publications have demonstrated promising results with algorithms in this task, although many are not yet clinically tested, and those that are do not cover the entire population. At Eugin, we have developed an AI model that has shown, in its development and preclinical validation phases, the ability to improve dosage in between 5.6% and 32% of cases.

¿How does this system work in practice?

The system consists of two parts: an online platform where the medical professional will input the necessary information for analysis (age, body mass index, and ovarian reserve data), and an AI model that autonomously analyzes this data, providing the recommended personalized hormonal medication dose.

Purpose of the study

Having completed preclinical validations and confirmed its theoretical effectiveness, we now need to determine for the first time whether the AI model developed performs at least as well as our ability to identify the ideal first dose of hormonal medication in practice. The objective of this study is to compare the number of mature eggs retrieved between a control group and a group where the first medication dose is assigned using the developed algorithm.

¿Why have you been selected to participate?

To conduct this pilot study, we require 236 participants randomized into two groups (control vs. study). You have been selected because you are undergoing your first in vitro fertilization (IVF) cycle with your own eggs. Additionally, you do not exhibit any exclusion criteria for this study: natural cycle treatment and/or use of hormonal medication not measured in international units (IU).

¿What does my participation involve?

If you agree to participate in this study, you will be randomly assigned to one of the two groups we are comparing: study treatment, where the first hormonal medication dose is assigned by the algorithm, or standard treatment (control group), where the first hormonal dose is assigned following the standard medical protocol. This means you have a 50% chance of receiving study treatment and a 50% chance of receiving standard treatment. The relevant results of your treatment will be analyzed, from the number of eggs retrieved to the pregnancy outcome after completing your IVF treatment. Your participation in this study will end when this final result is known. You will not be aware of your group classification (study or control).

To conduct the planned analyses in this study, we will need access to your medical history to collect your demographic and clinical data.

Your participation in this study is entirely voluntary. Your decision to participate or not will not impact the quality of your treatment or the care you receive. Additionally, you can withdraw from the study at any time by contacting the principal investigator of the study at the phone number or email provided at the end of this document, without the need for explanations and without any consequences. If you accept participation and can be included in this study, you will be offered compensation of 350 euros.

Risks and benefits of participation

The algorithm tested in this study has demonstrated, in its pre-clinical phases, greater accuracy in assigning the first dose of hormonal medication than current practice. Therefore, it is expected to behave similarly in its clinical phase, although there is no guarantee. This would potentially increase the chances of achieving an optimal number of mature eggs for your particular case. Conversely, assuming the algorithm is not more accurate than current practice, this potential increase may not occur, or there may be a lower or higher than expected recovery of eggs. A lower recovery of mature eggs could decrease the success chances of IVF treatment, and a higher recovery could increase the risk of ovarian hyperstimulation. In the latter case, the appropriate discharge protocol would be used to mitigate the hyperstimulation, avoiding, in the vast majority of cases, health problems but preventing a fresh embryo transfer. Embryos would be frozen and transferred in your next menstrual cycle. Given the conservative design of the algorithm and as evidenced in preclinical results, there is no expectation that the described risks will increase with its use compared to standard clinical practice. In accordance with Article 69 of Regulation 2017/745 for medical devices, the sponsor of this study has coverage for insurance or compensation for patients in the event of any unexpected serious adverse events.

In accordance with Article 7 of Law 14/2007 on Biomedical Research, your participation implies waiving any economic or other rights regarding the study's results.

Applicable regulations to this study

Following international ethical guidelines and current legislation, this study has been approved by the Ethics Committee for Drug Research at Eugin and the Spanish Agency of Medicines and Medical Devices. Your personal data will be strictly confidential, in accordance with Regulation (EU) 2016/679 of April 27 regarding the protection of individuals concerning the processing of personal data (GDPR) and Organic Law 3/2018 of December 5 on the protection of personal data and guarantee of digital rights (LOPDGDD). As the owner of your personal data, you have the right to access, modify, oppose, and delete your data, as well as to limit the processing of incorrect data, request a copy, or have your data transferred to a third party (portability). We remind you that your personal data cannot be deleted, even if you cease to participate in this study, to ensure the validity of the research; they will only be retained for the purposes of this study and will not be transferred to third parties except for legal obligations or the pursuit of a legitimate interest of the company, a third party, or yourself. In compliance with Article 89 of the GDPR, as well as the provisions of Additional Provision 17a of the LOPDGDD, your personal data collected for this study will be subject to adequate safeguards for your rights and freedoms and will therefore be identified by a code, excluding information that could identify you. Only the research team will be able to relate this data to you and your medical history. Therefore, your identity will not be disclosed to anyone else except to Health Authorities when required or in cases of medical emergency. Ethics Committees for Research, representatives of the Health Authority for inspection purposes, and personnel authorized by the Sponsor may only access

personal data to check personal data, study procedures, and compliance with good clinical practice standards (always maintaining the confidentiality of the information). The legal basis for processing will be the express and unequivocal consent provided by you through participation in this study.

In the current framework of biomedical research, the Investigator and the Sponsor are obligated to retain the data collected for this study for at least 25 years after its completion (15 years for clinical trials with medications). Subsequently, your personal information will only be retained by the center for your health care and by the sponsor for other scientific research purposes if you have given your consent.

In the event that we need to transfer your data outside the EU to entities in our group, service providers, or researchers collaborating with us, participant data will be subject to appropriate technical and organizational measures to ensure an adequate level of security according to the risk, including, among others: a) pseudonymization and encryption of personal data; b) the ability to guarantee the permanent confidentiality, integrity, availability, and resilience of processing systems and services; c) other additional safeguards such as contracts or other mechanisms defined by data protection authorities.

We inform you that you can contact the data protection officer of Clínica Eugin at dpo@eugin.es if you have any doubts, complaints, or need to exercise any of your mentioned rights. Likewise, we inform you of your right to address the Data Protection Agency if you are not satisfied.

We are at your disposal for any questions by phone (+34933221122) or email (mpopovic@eugin.es). Thanks to you, we can continue our research.

INFORMED CONSENT FORM

Randomized Study of an Artificial Intelligence-Based Medical Device for Selecting the First Dose of FSH for Ovarian Stimulation

Study Sponsored by Eugin Clinic and Conducted by Dr. Mina Popovic as Principal Investigator

I, (Name and surnames): ____

With ID/Passport number: ______________________________

Age: ________ and birth date: ______________________________

I declare that I have been informed about the current study and:

- Received sufficient information about the study.
- Read and understood the information sheet provided to me.
- Been able to ask questions about the study and my rights.
- Understood that my participation is voluntary.
- Understood that I can withdraw from the study at any time without having to provide explanations, and this will not affect my medical care.
- Understood that my participation in the study does not pose any harm to my health.

Been informed that personal data will be protected, and the results of my personal evaluation will be strictly confidential.

Therefore, I give my consent to participate in this study.

Participant's signature<br><br>Name, surnames<br><br>Date: ____________________________	Informing physician's signature<br><br>Name, surnames, registration number<br><br>Date: ____________________________
